# [^18^F]FDG and [^18^F]FES positron emission tomography for disease monitoring and assessment of anti-hormonal treatment eligibility in granulosa cell tumors of the ovary

**DOI:** 10.18632/oncotarget.27925

**Published:** 2021-03-30

**Authors:** Joline F. Roze, Hannah S. van Meurs, Glen R. Monroe, Wouter B. Veldhuis, Luc R.C.W. van Lonkhuijzen, Roel J. Bennink, Jolijn W. Groeneweg, Petronella O. Witteveen, Geertruida N. Jonges, Ronald P. Zweemer, Arthur J.A.T. Braat

**Affiliations:** ^1^Department of Gynecological Oncology, University Medical Center Utrecht, Utrecht University, Utrecht, The Netherlands; ^2^Department of Gynecological Oncology, Amsterdam University Medical Center, Amsterdam, The Netherlands; ^3^Department of Radiology, University Medical Center Utrecht, Utrecht University, Utrecht, The Netherlands; ^4^Department of Radiology and Nuclear Medicine, Amsterdam University Medical Center, Amsterdam, The Netherlands; ^5^Department of Medical Oncology, University Medical Center Utrecht, Utrecht University, Utrecht, The Netherlands; ^6^Department of Pathology, University Medical Center Utrecht, Utrecht University, Utrecht, The Netherlands; ^7^Department of Radiology and Nuclear Medicine, University Medical Center Utrecht, Utrecht University, Utrecht, The Netherlands

**Keywords:** positron emission tomography (PET), 18F-fluoroestradiol (^18^F-FES), 18F-fluoro-deoxyglucose (^18^F-FDG), granulosa cell tumors (GCTs), hormone receptors

## Abstract

Purpose: Adult granulosa cell tumors (AGCTs) of the ovary represent a rare malignancy in which timing and choice of treatment is a clinical challenge. This study investigates the value of FDG-PET/CT and FES-PET/CT in monitoring recurrent AGCTs and assessing eligibility for anti-hormonal treatment.

Materials and Methods: We evaluated 22 PET/CTs from recurrent AGCT patients to determine tumor FDG (*n* = 16) and FES (*n* = 6) uptake by qualitative and quantitative analysis. We included all consecutive patients from two tertiary hospitals between 2003-2020. Expression of ERα and ERβ and mitoses per 2 mm^2^ were determined by immunohistochemistry and compared to FES and FDG uptake, respectively.

Results: Qualitative assessment showed low-to-moderate FDG uptake in most patients (14/16), and intense uptake in 2/16. One patient with intense tumor FDG uptake had a high mitotic rate (18 per 2 mm^2^) Two out of six patients showed FES uptake on PET/CT at qualitative analysis. Lesion-based quantitative assessment showed a mean SUV_max_ of 2.4 (± 0.9) on FDG-PET/CT and mean SUV_max_ of 1.7 (± 0.5) on FES-PET/CT. Within patients, expression of ERα and ERβ varied and did not seem to correspond with FES uptake. In one FES positive patient, tumor locations with FES uptake remained stable or decreased in size during anti-hormonal treatment, while all FES negative locations progressed.

Conclusions: This study shows that in AGCTs, FDG uptake is limited and therefore FDG-PET/CT is not advised. FES-PET/CT may be useful to non-invasively capture the estrogen receptor expression of separate tumor lesions and thus assess the potential eligibility for hormone treatment in AGCT patients.

## INTRODUCTION

Granulosa cell tumors are a well-defined ovarian cancer subtype, responsible for 2-5% of ovarian malignancies with an annual incidence of 0.6–1.0 per 100.000 women worldwide [1–4]. The tumor arises from the estrogen producing granulosa cells present in the ovarian stroma. Adult (95%) and juvenile (5%) subtypes can be distinguished based on clinical and histopathological characteristics. The juvenile subtype generally occurs in prepubertal girls and young women, whereas the adult type has its peak incidence between 50-55 years [4].

Adult granulosa cell tumors (AGCTs) harbor a specific missense mutation in *FOXL2* in approximately 95% of cases [5, 6]. Continuous exposure to tumor-derived estrogen can cause endometrial proliferation. As a result, 6% of patients have concomitant endometrial cancer at diagnosis [2]. Common symptoms of AGCTs include abnormal vaginal bleeding and abdominal pain. Surgery is the mainstay of treatment throughout the disease course, due to generally limited effects of systemic treatments such as chemotherapy and hormone therapy [5, 7]. Recurrences occur in approximately 50% of patients and often require repeated debulking surgeries. Of women with recurrent disease, 50–80% ultimately succumb to their disease [5, 8, 9].

Surgical treatment has its limitations, as risks of surgery increase with subsequent debulking procedures. Therefore, when a recurrence is detected, it can be justified to opt for watchful waiting with frequent disease monitoring while patients retain a good quality of life. Vice versa, surgery should be performed when all tumor deposits can still be completely removed. This results in a limited therapeutic window, making the timing of surgical resection a clinical challenge. Imaging tools that are able to measure disease activity, can potentially help to optimize the timing of surgery.

Fluor-18-deoxyglucose uptake on positron emission tomography combined with computed tomography (FDG-PET/CT) defines the metabolic activity of cells and has proven to be useful for staging and detection of recurrence in many cancer types. To date, anecdotal case reports with a total of five patients describe the use of FDG-PET in granulosa cell tumors, and show conflicting results [10–13]. These studies found no FDG uptake in two patients, moderate uptake in two patients and intense FDG uptake in one patient with a bone metastasis.

AGCTs commonly express the estrogen receptors alpha (ERα, 30–66%) and beta (ERβ, 94–100%) [14–17]. Therefore, anti-hormonal treatment is thought to be a potentially effective treatment strategy. A previous study showed that anti-estrogen treatment can decrease tumor load in a subset (*n* = 4; 18%) of 22 AGCT patients [7]. Anti-hormonal treatment is generally well tolerated and can be used continuously for many years. Nevertheless, it remains difficult to determine the treatment of choice and to identify patients that may benefit from this treatment. Recently, PET using the 16α-18F-fluoro-17β-estradiol FES tracer became available to noninvasively assess ER expression. This novel technique is used in hormone receptor positive tumors, such as breast cancer, to identify candidates for anti-hormonal treatment [18]. In these cancers, tamoxifen (selective estrogen receptor modulator), anastrazole and letrozole (aromatase inhibitors) and fulvestrant (estrogen receptor antagonist), have been widely used and show comparable efficacy [19, 20]. The use of FES-PET/CT has not yet been evaluated in AGCTs.

This study investigates the value of FDG-PET/CT for disease monitoring and FES-PET/CT for indicating anti-hormonal treatment eligibility in AGCT patients.

## RESULTS

**Figure 1:**
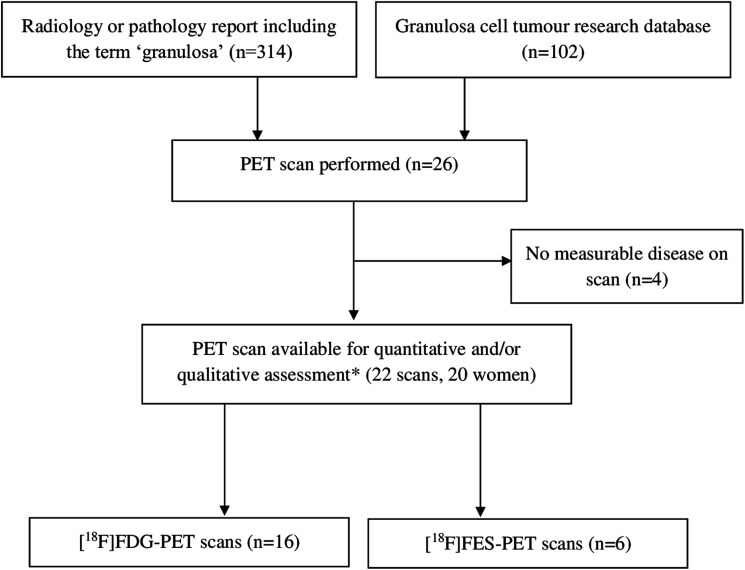
PET scan selection. ^*^Three scans were eligible only for qualitative assessment.

A total of 26 PET/CTs was performed, of which 19 were eligible for both qualitative and quantitative assessment, and three for qualitative assessment only (22 scans in 20 women, Figure 1). Four out of 26 scans were excluded due to a lack of measurable disease. All scans were performed between June 2003 and May 2020 in two academic hospitals (UMC Utrecht and Amsterdam UMC, location AMC). The studied PET/CTs included 16 FDG-PET/CTs and six FES-PET/CTs. FDG-PET/CTs were performed for detection of a recurrence (15/16), or to assess response to chemotherapy (1/16). All FES-PET/CTs were prospectively and consecutively performed in recurrent AGCTs to evaluate tumor FES uptake prior to hormone treatment (4/6) or to assess disease load prior to surgery (2/6). Baseline characteristics and imaging parameters are presented in Table 1.

**Table 1:** Baseline characteristics and imaging parameters

		FDG	FES
**Number of PET/CTs**		16	6
		*Median (range)*	*Median (range)*
**Age**		67 (39–74)	69 (62–76)
**Time since diagnosis (years)**		11 (2–41)	12 (6–28)
**Clinical indication**	Detection of recurrence	15 (94%)	-
	Assess disease load prior to surgery	-	2 (33%)
	Assess FES uptake prior to anti-hormonal treatment	-	4 (67%)
	Assess response to chemotherapeutic treatment	1 (6%)	-
**Serum glucose level in mmol/L**		5.2 (4.0–5.9)	NA
**IA in MBq**		176 (79–325)	190 (129–202)
**IA in MBq/kg**		2.72 (1.46–5.2)	2.18 (1.55–3.39)
**Acquisition time post-injection in minutes**		60 (43–78)	63 (52–96)^*^

^*^One patient had a delay in imaging and was scanned at 96 minutes post-injection. All other patients were scanned with an interval of 52–64 minutes. Legend: IA = injected activity, NA = not applicable.

Qualitative assessment of FDG-PET/CTs showed no uptake in three patients (19%), moderate uptake in eleven patients (69%), and intense uptake in two patients (13%), in previously known metastases found on CECT (Table 2). In one patient, an additional moderate FDG-avid liver metastasis was detected by FDG-PET/CT after the initial CECT. In all other cases, FDG-PET/CT did not detect any additional metastases compared to CECT. FES-PET/CT imaging showed no uptake in one out of six patients (17%), low uptake in three out of six patients (50%) and moderate uptake in the remaining two patients (33%). No additional metastatic lesions were detected by FES-PET/CT compared to those identified on the initial CECT.

**Table 2:** Qualitative assessment of FDG-PET/CT and FES-PET/CT

	FDG-PET/CT	FES-PET/CT
**Patient based assessment**		
Number of PET/CTs	16	6
Negative or low uptake	3	4
Moderate uptake	11	2
Intense uptake	2	0
**Metastatic lesions**		
Number of lesions	41	22
Suspected solid (enhancing) on CECT	31	8
Suspected cystic (non-enhancing) on CECT	10	14

Quantitative assessment of all visually detectable lesions (32) on FDG-PET/CT showed a mean SUV_max_, SUV_mean_ and SUV_peak_ of 2.4, 1.4 and 1.9, which was higher than the mean blood pool SUV_mean_ (1.2), however not markedly increased as compared to the mean liver SUV_max_ (2.2) or liver SUV_mean_ (1.6) (Table 3). Even though most patients were found to have solid metastases on CECT (24/32 lesions), no difference was noted in FDG-avidity as compared to cystic lesions (8/32 lesions; Table 3). Quantitative assessment of all visually detectable lesions (12) on FES-PET/CT showed a mean SUV_max_, SUV_mean_ and SUV_peak_ of 1.7, 1.0 and 1.4, all within the range of the mean blood pool SUV_mean_ (1.4 ± 1.2 SD). In agreement with the FDG-PET/CT findings, there was no difference in FES uptake between suggested solid (7/12 lesions) and suggested cystic (5/12 lesions) metastases on CECT (Table 3).

**Table 3:** Quantitative assessment of FDG-PET/CT and FES-PET/CT positive lesions by visual assessment

	FDG-PET/CT			FES-PET/CT		
General parameters (patient based)						
Number of scans	13			6		
Mean SUV_mean_ BP (±SD)	1.2 (±0.4)			1.4 (±1.2)		
Mean SUV_max_ BP (±SD)	1.5 (±0.5)			1.7 (±1.2)		
Mean SUV_mean_ liver (±SD)	1.6 (±0.4)			9.6 (±4.4)		
Mean SUV_max_ liver (±SD)	2.2 (±0.6)			18.3 (±16.1)		
**Metastatic lesion measurements (lesion based)**						
	All	*Solid*	*Cystic*	All	*Solid*	*Cystic*
Number of lesions	32	*24*	*8*	12	*7*	*5*
Mean SUV_max_ (±SD)	2.4 (±0.9)	*2.5 (±0.9)*	*1.8 (±0.8)*	1.7 (±0.5)	*1.8 (±0.5)*	*1.6 (±0.4)*
Mean SUV_mean_ (±SD)	1.4 (±0.6)	*1.5 (±0.5)*	*1.2 (±0.5)*	1.0 (±0.3)	*1.0 (±0.3)*	*1.0 (±0.3)*
Mean SUV_peak_ (±SD)	1.9 (±0.8)	*2.0 (±0.8)*	*1.5 (±0.6)*	1.4 (±0.4)	*1.4 (±0.4)*	*1.5 (±0.4)*
No. SUV_mean_ > BP SUV_mean_ (%)	18 (57%)	*15 (63%)*	*3 (38%)*	5 (38%)	*3 (38%)*	*2 (40%)*
No. SUV_mean_ > liver SUV_mean_ (%)	5 (16%)	*4 (17%)*	*1 (12%)*	*	*	*
No. SUV_max_ > liver SUV_max_ (%)	16 (50%)	*13 (54%)*	*3 (38%)*	*	*	*

Legend: SD = standard deviation, BP = blood pool. *for FES-PET/CT this measurement is not applicable, because of high physiological biliary excretion of the tracer.

**Figure 2:**
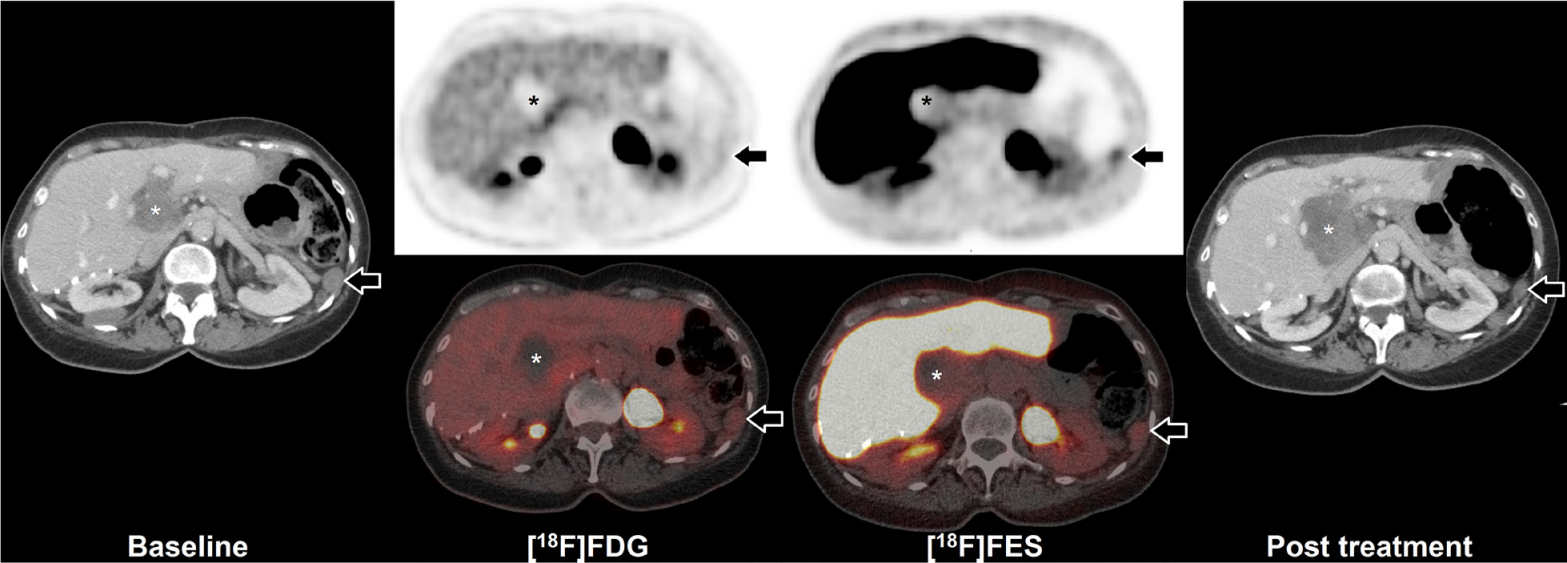
FES-PET and response to hormone treatment. Example of FDG and FES in a 64 year old woman with peritoneal and hepatic metastases of AGCT. Baseline CECT (left) shows peritoneal disease (arrow) and hepatic disease (asterisk). Additional FDG-PET/CT shows low uptake in the peritoneal disease, whilst FES shows moderate uptake (arrows). The majority of liver hilum lesion accumulates neither FDG nor FES (asterisks). After initiation of hormonal treatment with letrozole for six months, follow-up CECT (right) showed partial regression of the FES positive peritoneal lesion (from 23 mm to 17 mm maximal diameter; arrows), whilst the FES negative hepatic lesion showed progression (from 50 mm to 65 mm maximal diameter; asterisks).

**Figure 3:**
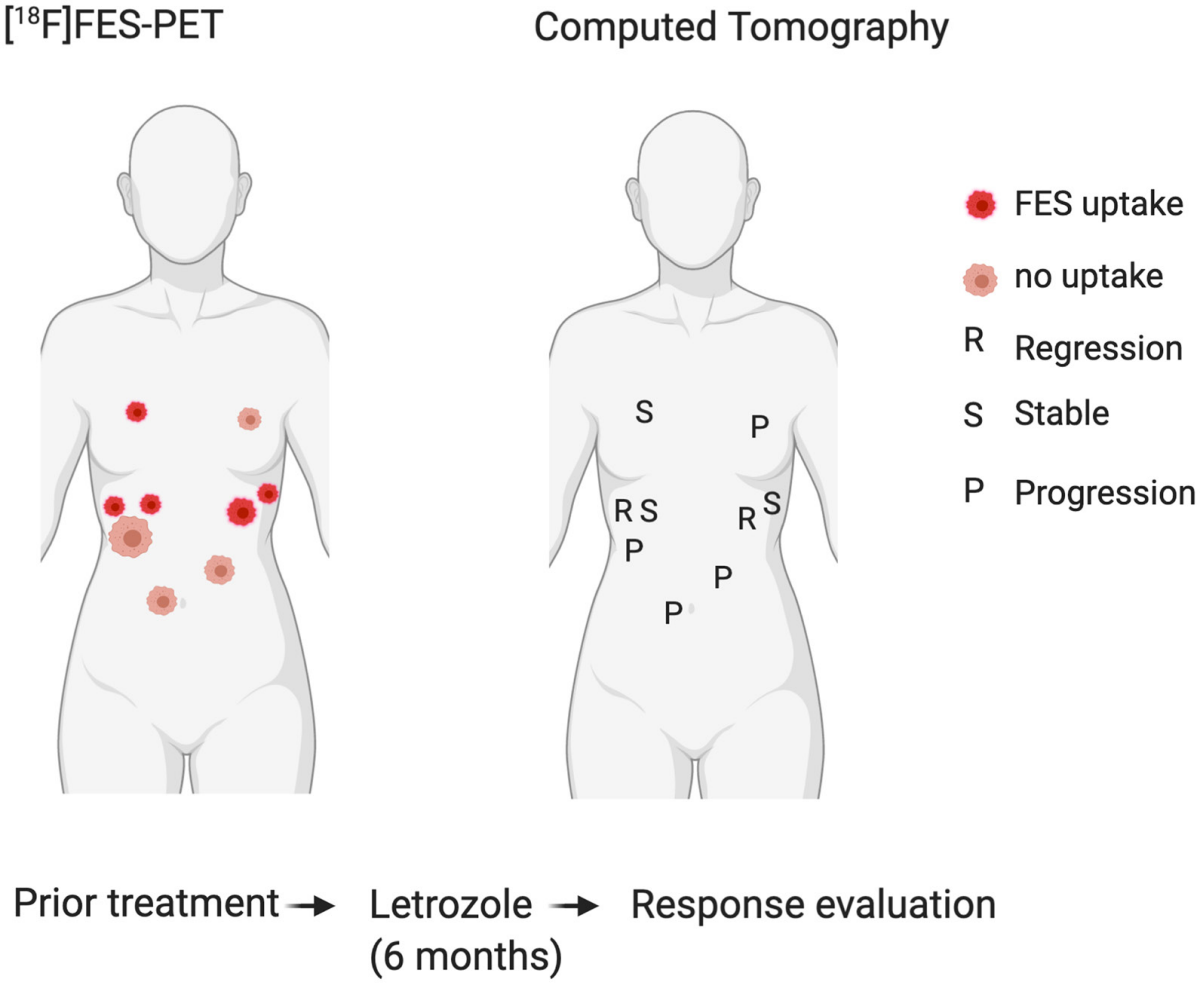
Correlation of FES positive and negative tumour locations (left) with response to hormone treatment (right). All FES negative lesions showed progression after six months hormone treatment, whereas all FES positive lesions showed stable disease or regression.

Four patients received FES-PET/CT to assess FES tumor uptake prior to anti-hormonal treatment, of which one had multiple FES positive lesions (Table 1). This patient underwent both FDG and FES-PET/CT imaging prior to anti-hormonal treatment. PET/CT showed low FDG uptake and moderate FES uptake of a peritoneal tumor lesion (Figure 2). After anti-hormonal treatment with letrozole for six months, a follow-up CECT showed progression of all FES negative lesions, whereas all FES positive lesions showed stable disease or regression (Figure 3).

The other three patients who received FES-PET/CT prior to anti-hormonal treatment did not have FES tumor uptake. Two of them were treated with tamoxifen and one patient refrained from anti-hormonal treatment. In these patients, the response to tamoxifen was evaluated on CT scan after four to six months treatment and showed disease progression and a newly developed peritoneal lesion in both patients.

Evaluation of mitotic activity by immunohistochemistry was performed on available tissue of 15/16 (94%) patients in the FDG-PET group. ER expression was assessed for all patients in the FES-PET group (Supplementary Table 1). The number of mitoses per 2 mm^2^ was < 10 for all samples (median 5, range 1–18) except for one showing 18 mitoses per 2 mm^2^, which had intense uptake on FDG-PET. We did not detect a correlation between ER expression and FES uptake on PET/CT. ERα expression was positive (≥ 5%) in 5/6 patients (83%) and ERβ in all six patients. Most patients had either predominantly ERα or predominantly ERβ receptor expression.

## DISCUSSION

This is the first study to investigate the value of FDG-PET/CT in monitoring recurrent AGCTs and FES-PET/CT to assess eligibility for anti-hormonal treatment. FDG-PET/CT showed low to moderate uptake in most patients (14/16 scans) and identified only one additional tumor location as compared to CT scan. FES uptake on PET/CT was present in 33% (2/6) of the patients. One of these patients also had FES negative lesions, which progressed after six months of anti-hormonal treatment, whereas all FES positive lesions showed stable disease or regression. In this patient, PET/CT using the FES tracer captured the intra-patient tumor heterogeneity and FES uptake correlated with the individual tumor response to hormone treatment. Moreover, there was also a clear clinical correlation for the patients without FES tumor uptake, as the CT scans showed progressive disease after anti-hormonal treatment with tamoxifen.

Although four out of five AGCT cases previously described in case reports showed low to moderate FDG avidity, contradictory results and lack of FDG-PET/CT series emphasized the need for further investigation. The current study shows that FDG avidity of AGCTs is low or moderate and result in low detection rates of (metastatic) disease by FDG-PET only. In particular peritoneal metastases may be difficult to distinguish from physiologic bowel uptake. Also, FDG-PET/CT did not identify additional metastasis as compared to CECT. Imaging by FDG-PET may therefore not be helpful in monitoring AGCTs. However, one of two patients with intense FDG tumor uptake and with the highest mitotic rate (18 per 2 mm^2^) harbored an AGCT with a *TP53* mutation, which has been described in a subset of AGCT patients (9–11%). This mutation may explain the FDG uptake in this patient, since TP53 mutant AGCTs are associated with a higher tumor mutational burden, mitotic rate and metabolic activity [21]. If this finding can be confirmed in a larger subset of AGCT patients with TP53 mutations, FDG-PET/CT may be of value in this small subpopulation.

Previous studies showed that FES uptake corresponds with ER expression on immunohistochemistry in breast cancer and uterine cancer [22, 23]. Compared to immunohistochemical staining, FES-PET has a sensitivity of 0.81 and specificity of 0.86 in breast carcinoma studies [24]. We could not confirm this correlation in AGCTs, potentially due to the small sample size of the study, the time interval between tissue withdrawal and the FES-PET/CT, and varying ER expression levels between tumor lesions. Heterogeneous ER expression has also been detected in breast cancer and discordant expression between primary tumors and metastasis is seen in up to 40% of the patients [18, 25]. Compared to immunohistochemistry, FES-PET/CT is a noninvasive method that provides a comprehensive overview of all existing tumor locations and the ER expression of individual metastatic lesions. Intra-patient tumor heterogeneity is common in AGCTs [21], making the comparison between ER expression on older tissue samples and FES uptake more difficult. Additionally, the affinity of FES for the ERα is 6.3 times higher than that for the ERβ. FES-PET/CT imaging may therefore better reflect ERα expression, while ERβ overexpression is more common in AGCTs (Supplementary Table 1) [18]. Nevertheless, this study demonstrates that FES-PET/CT can visualize estrogen receptor binding in AGCTs and that FES tumor uptake correlates with the response to anti-hormonal treatment in a single case. Therefore, FES-PET/CT could help to provide a rationale for anti-hormonal treatment. In addition, absent tumor FES uptake in AGCT lesions may predict failure of hormonal therapy or resistant locations in patients, as illustrated by one of our cases.

The current study has a few limitations. Both in case reports and in case series, results may be influenced by selection bias. In this study, patients were not randomly selected to undergo PET scanning and most of them had a long history of AGCT recurrences to which different treatment strategies were applied. This has led to a heterogeneous study population. It is uncertain whether this may have influenced the outcome of the PET scans, as FES uptake may potentially be higher in primary disease than in recurrent lesions. Concerning the quantitative analysis of FES, no validated cutoff for FES PET in gynaecological tumors in known in literature. However, an optimal cutoff is suggested in breast cancer (SUV_max_ of 1.54 for FES PET), which is in line with the results of our visual and quantitative assessment [18, 26, 27].

To our knowledge, this study is the first to investigate the value of a targeted hormone tracer in AGCTs. Nuclear agents binding to other hormone receptors expressed by AGCTs, such as AMH and the progesterone receptor, could also be good candidates for targeted PET scanning.

It remains a clinical challenge to establish the optimal timing of treatment for AGCT recurrences. Besides PET-CT, other diagnostics such as detection of circulating tumor DNA in plasma, are currently being investigated for disease monitoring and estimation of disease activity [28, 29]. Given the low incidence of this disease, performing prospective trials in AGCT is difficult. Future prospective research on FES-PET/CT could elucidate whether this imaging tool can be used to predict the response to hormonal treatment in AGCT patients.

## MATERIALS AND METHODS

### Patients

To identify the available PET scans, all radiology and pathology reports of two academic hospitals from 2000–2020 including the term ‘granulosa’ were retrieved (Figure 1). Additionally, we searched for patients who underwent PET/CT evaluation in our AGCT research database. This database currently contains 102 patients with a pathologically confirmed AGCT, from six hospitals in the Netherlands, included between 2017 and 2020. All FDG-PET scans with measurable disease from patients with a histologically confirmed AGCT were included. In addition, six FES-PET/CTs were prospectively performed for clinical purposes and included in this study (Table 1). Ethical approval was obtained by the Institutional Review Board of the University Medical Center Utrecht. All participants provided written informed consent for the use of their clinical data. Statistical analyses were performed using SPSS v.25.0 (IBM Corp., Armonk, NY, USA). Patient characteristics are described as median and range, and all imaging parameters as mean and standard deviation (SD).

### FDG-PET/CT

Injected FDG activity was approximately 2–3 MBq/kg for all PET/CTs. Due to the multicenter retrospective nature of this study and the long time period of inclusion for this rare malignancy, no standardized methods for FDG-PET/CT reconstructions were available before 2010, limiting the reproducibility of standardized uptake value (SUV) measurements. From 2010 and onwards, after the introduction of the EARL-criteria in 2010, all FDG-PET/CTs were acquired and reconstructed according to these international guidelines [30].

### FES-PET/CT

Images were acquired from thighs to skull vertex on a single PET/CT scanner (Biograph mCT, Siemens, Erlangen, Germany) and approximately 60 minutes after intravenous injection of 200 MBq FES. A low dose CT was performed directly following PET acquisition. Images were acquired according to the European Association of Nuclear Medicine (EANM) criteria, a.k.a. EARL-reconstructions, with the following parameters: PET with time-of-flight and point spread function reconstruction, 4 iterations, 21 subsets, with a filter of 7.5 mm full width at half maximum [30].

### Image analysis

All retrospectively gathered and available FDG-PET/CT and prospective FES-PET/CT images were centrally reviewed and analyzed, along with contrast enhanced CTs (CECT) of the thorax, abdomen and pelvis, by a nuclear medicine physician (AJATB, > 5 years of experience). Besides assessment of the number and location of metastatic lesions and CECT, all suspected metastatic lesions were divided between solid (predominantly enhancing) or cystic/necrotic (predominantly non-enhancing) lesions. Qualitative assessment of all available imaging included a blinded assessment of all PET/CT imaging, and comparison with a prior CECT. Additionally, all PET/CT imaging was scored patient based according to a visual scale (negative, low, moderate or intense), with a maximum of five lesions per patient (Supplementary Table 2).

Quantitative assessment of all FDG- and FES-PET/CTs was performed using software package Syngo.via (Siemens, Erlangen, Germany). As a reference, both liver uptake (3 cm^3^ VOI in the right liver lobe) and blood pool measurements (cylindrical volume in the descending thoracic aorta) were acquired. All known lesions detected on contrast enhanced diagnostic CT and moderate or intense positive according to the qualitative assessment were measured on the PET/CTs (lesion based, with a maximum of five lesions per patient) (Supplemetnary Table 2). Spherical volumes of interest (VOI’s) were manually drawn, visually matching and including the entire metastasis as detected on CECT (contrast enhanced CT). Standardized uptake values (SUV) were calculated according to the lean body mass method, in line with PERCIST recommendations. All measurements are reported as SUV_max_, SUV_peak_ and SUV_mean_.

### Immunohistochemistry

Immunohistochemistry was performed in available surgical specimens, to assess expression of the estrogen receptors (ERα and ERβ rabbit antibody, Ventana RTU dilution, Roche), and ER expression was evaluated consistently by a single pathologist and scored as a percentage. The number of mitoses was counted per 2mm^2^. The correlation between FES uptake (SUV_max_) and ER status and FDG uptake (SUV_max_) and number of mitoses, respectively, was assessed and visualized in a scatterplot.

### Informed consent

All patients provided written informed consent for the use of their clinical data and publication of results. Ethical approval was obtained by the Institutional Review Board of the University Medical Centre Utrecht. Clinical data were acquired from patient reports.

## CONCLUSIONS

This study shows that FDG uptake by AGCTs is low-moderate and FDG-PET/CT may not be helpful in monitoring AGCTs. Our FDG-PET/CT findings are in corroboration with previous reports and provide a possible explanation for incidental patients with high FDG tumor uptake (i.e., high mitotic index due to a *TP53* mutation). Furthermore, FES uptake was seen in four AGCT patients and correlated with the response to anti-hormonal treatment. FES positive tumors remained stable or decreased in size as FES negative tumors progressed after anti-hormonal treatment. Therefore, FES-PET/CT may be useful to assess the potential eligibility for anti-hormonal treatment in AGCT patients by noninvasively capturing the estrogen receptor expression levels of all separate tumor lesions.

## SUPPLEMENTARY MATERIALS


