# Supplementary material for: [18F]FDG and [18F]FES positron emission tomography for disease monitoring and assessment of anti-hormonal treatment eligibility in granulosa cell tumors of the ovary
Source: Oncotarget. 2021 Mar 30;12(7):665–73. doi: 10.18632/oncotarget.27925 (PMC8021033; doi:10.18632/oncotarget.27925)
Supplement: Supplementary file 1 [file oncotarget-12-665-s001.pdf]

[^{18}F]FDG and [^{18}F]FES positron emission tomography for disease monitoring and assessment of anti-hormonal treatment eligibility in granulosa cell tumors of the ovary

SUPPLEMENTARY MATERIALS

Supplementary Table 1: Correlation between PET tracer uptake, cell proliferation and hormone receptor expression

PET-ID	Year scan	SUV_{max}	Qualitative assessment	Immunohistochemistry	
**FDG**				**Mitoses per 2 mm²**	
P1	2009	1,82	Moderate	NA	
P2	2013	1,34	Moderate	7	
P3	2008	Negative	Negative	5	
P4	2010	Negative	Negative	5	
P5	2011	1,71	Moderate	5	
P6	2011	0,81	Moderate	2	
P7	2003	NA	Intense	18	
P8	2006	NA	Moderate	3	
P9	2013	2,53	Moderate	3	
P10	2007	NA	Moderate	9	
P11a*	2019	1,76	Moderate	9	
P12	2018	1,9	Moderate	2	
P13	2018	3,57	Intense	9	
P14	2018	Negative	Negative	2	
P16a*	2014	2,68	Moderate	9	
P18	2020	2,94	Moderate	1	
**FES**				**ERα**	**ERβ**
P11b	2019	1,3	Moderate	10%	67%
P15	2019	2,03	Low**	85%	24%
P16b	2020	1,41	Low	5%	90%
P17	2020	2,36	Moderate	50%	100%
P19	2020	Negative	Negative	80%	90%
P20	2020	1,84	Low	0%	100%

Correlation between FDG uptake and cell proliferation, and FES uptake and estrogen receptor expression, respectively. *Patient 11 and 16 received both FDG-PET/CT and FES-PET/CT. **Although the overall visual qualitative assessment in this patient is low uptake, just one lesion was positive (resulting in an SUVmax of 2,03). NA: not applicable.

Supplementary Table 2: Qualitative assessment; visual score scale in at least one suspected metastasis

Visual reference	Conclusion
No uptake	Negative
Less or equal to bloodpool	Low
More than bloodpool and equal to liver	Moderate
Obviously more than liver*	Intense

*for FES-PET/CT this measurement is not applicable, because of high physiological biliary excretion of the tracer.
